# Surgical correction of stenotic nares using a single pedicle advancement flap technique in three brachycephalic cats

**DOI:** 10.1002/vms3.1309

**Published:** 2023-10-28

**Authors:** Negin Rahimdoust Mozhdehi, Hamideh Salari Sedigh, Hossein Kazemi Mehrjerdi

**Affiliations:** ^1^ Faculty of Veterinary Medicine Department of Clinical Sciences Ferdowsi University of Mashhad Mashhad Iran

**Keywords:** airway obstruction, brachycephalic, corrective surgery, nares stenosis

## Abstract

Brachycephalic obstructive airway syndrome (BOAS) comprises a group of anatomical upper respiratory tract abnormalities that collectively result in various degrees of upper respiratory tract obstruction. Stenotic nares is a common feature of BOAS, and in dogs, the main cause is axial deviation of the alar cartilage. In contrast, narrowing of the nares in cats is predominantly the result of a redundant skin fold at the junction of the ventral floor of the nostrils and the haired skin of the lip. Three brachycephalic cats with inspiratory obstruction were referred to the surgery department of the Veterinary Teaching Hospital of Ferdowsi University of Mashhad, Iran. The predominant cause of obstruction was nostril stenosis due to the presence of redundant skin on the ventral floor of the nares. All three cats underwent surgical correction using a single pedicle advancement flap technique, which was first described by Berns et al. (2020). All three cats had positive outcomes, with no surgical complications and no episodes of respiratory distress reported within a 9‐month follow‐up. Appropriate surgical treatment of feline patients with stenotic nares can result in good long‐term outcomes.

## Graphical abstract

The single pedicle advancement flap technique was performed on three brachycephalic cats suffering from nostril stenosis, which was due to the presence of redundant skin in the ventral floor of their nares. By the 9‐month follow‐up following the procedure, no episodes of respiratory distress and no surgical complications were reported.

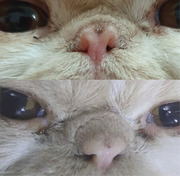

## 1 INTRODUCTION

Brachycephalic breeds such as English and French bulldogs, pugs, Boston Terriers and Persian cats have a shorter and wider skull in comparison to mesaticephalic and dolichicephalic breeds (Chanel N. Berns et al., 2020; Dupré and Heidenreich, 2016). In proportion to the whole body size, these breeds have a normal lower jaw, but the upper jaw and longitudinal skull axis are shortened (Lodato & Hedlund, 2012a). This anatomical difference results in a narrowed upper respiratory tract lumen, and consequently, airway resistance is increased (Lodato & Hedlund, 2012b).

Brachycephalic obstructive airway syndrome (BOAS) refers to a group of abnormalities that collectively result in upper airway obstruction. The abnormalities include: (1) Skull conformation anomalies such as abnormal nasopharyngeal turbinates, nasal turbinate extension into the nasopharynx or dorsal rotation of the maxillary bone. (2) Soft tissue changes such as stenotic nares or an elongated, hyperplastic soft palate. (3) Laryngeal, tracheal and bronchial anomalies such as everted laryngeal saccules, laryngeal collapse, tracheal hypoplasia or bronchial collapse (Lodato & Hedlund, 2012). These culminate in variable clinical signs, including exercise and heat intolerance, stertor/stridor, dyspnea, cyanosis and syncope, depending on the number and severity of abnormalities present (Anagrius et al., 2021; Chanel N. Berns et al., 2020).

Most research around BOAS has been concentrated on dogs, but there is an assumption that the findings may also be applicable to cats. Indeed, studies of brachycephalic cats have shown that there is a correlation between a shorter nose and owner‐perceived increased respiratory noise and breathing difficulties (Anagrius et al., 2021).

The predominant BOAS‐related problem described in brachycephalic cats is stenotic nares. Unlike in dogs, the other aforementioned abnormalities are rarely observed (Chanel N. Berns et al., 2020), although eversion of the saccules and tonsils has been reported in cats (Anagrius et al., 2021) as has soft palate elongation in a Persian cat presenting with respiratory distress and pulmonary edema (Corgozinho et al., 2012).

It has been noted that dogs with clinically significant stenotic nares have an axial deviation of their alar fold (Berns et al. 2020). In contrast, redundant skin at the junction of the ventral floor of the nares and haired skin of the lip is the most common cause in cats, and axial deviation of the alar wings is less significant (Chanel N. Berns et al., 2020). The aim of this study was to further assess the use of the single pedicle advancement flap technique previously described by Berns *et al.*, for the treatment of feline stenotic nares (Chanel N. Berns et al., 2020).

## 2 MATERIAL AND METHODS

Three brachycephalic cats with a diagnosis of BOAS were referred to the surgery department of the Veterinary Teaching Hospital of Ferdowsi University of Mashhad, Iran, in 2020. Their medical records, including physical examinations, blood results, previous medications, radiological evaluations of the chest and the upper respiratory tract, laryngeal and oral examinations and the final diagnosis were reviewed. Stenotic nares were considered to be the predominant clinical problem in all three cats and all underwent surgical treatment as described by Berns et al. (2020).

### 2.1 Case description

**FIGURE 1:**
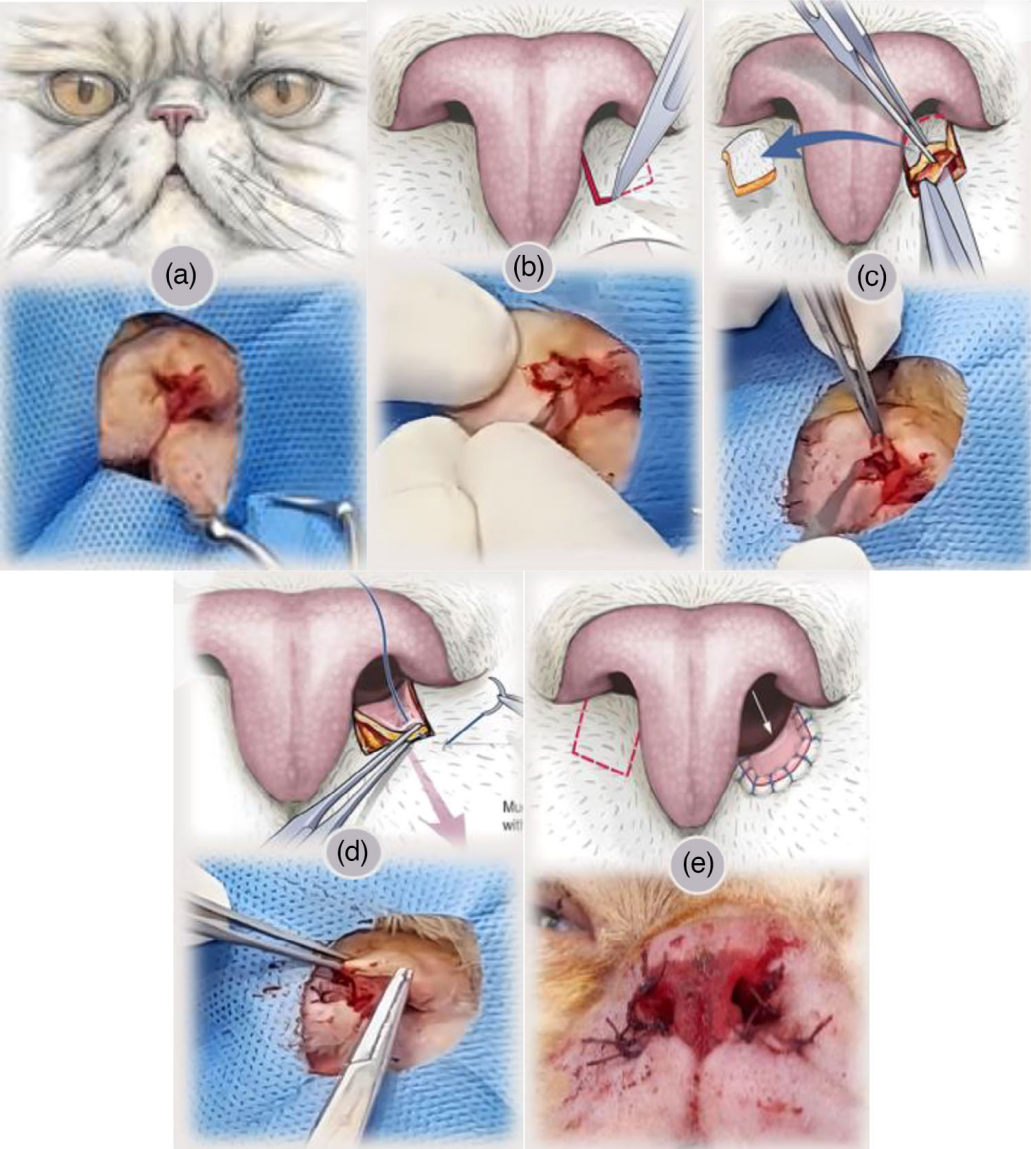
(a) Illustration of a cat with stenotic nares due to redundant skin on ventral floor. (b) An incision is made to create a mucosal flap. (c) The flap pedicle is expanded and the redundant skin is excised. (d) The flap is sutured with a single interrupted suture pattern. (e) The procedure is repeated for the contralateral nares (Schematic images were obtained from Berns et al., 2020).

Prior to general anesthesia, the cats were premedicated with acepromazine 10 mg/mL (0.1 mg/kg IM), and 20 min later, they were induced with Ketamine 10% (Bermer Pharma GMBH) (6 mg/kg IV) in combination with midazolam (Caspian Tamin, Iran) (0.25 mL/kg IV). Following intubation, anaesthesia was maintained using isoflurane (Piramal Critical Care, USA). The patients were positioned in sternal recumbency, and the surgical site was clipped and cleaned (Figure 1a). Using the number 11 scalpel blade, a single pedicle advancement flap was made bilaterally with a width equal to that of the ventral floor of the nares (Figure 1b). The flap was then advanced rostrocaudally. Its pedicle was extended to the skin of the upper lip, and the redundant facial skin was excised (Figure 1c). Finally, the flap was anchored with a simple interrupted suture pattern in a single layer using 5‐0 Polyglactin 910 suture material (Figure 1d). The procedure was performed on both sides (Figure 1e). In one of the patients, alarplasty was performed concurrently.

## 3 RESULTS

The three patients comprised a 4‐year‐old castrated male Persian flat‐faced cat, a 1.5‐year‐old spayed female Persian cat and a 2‐ year‐old intact male Chinchilla‐Scottish cat. All presented with a history of chronic dyspnea, nasal discharge, stertor/stridor, restlessness and decreased activity that had been recurrent following medical therapy. According to their clinical history, medications had been different for each patient, but all of them had received broad‐spectrum antibiotics for periods of time. One of these cats had been suffering from respiratory problems for approximately 1 year, and multiple medication regimes had been prescribed. In all three cats, physical examination confirmed upper respiratory tract obstruction and stenotic nares with no other clinically significant abnormalities. The results of previous routine blood screens were reported as unremarkable.

Radiography comprising lateral and ventrodorsal views of the thorax, and lateral and ventrodorsal views of the upper respiratory tract, had identified no evidence of concurrent tracheal or pulmonary pathology. Additionally, oral and laryngeal examinations under sedation, had identified no evidence of soft palate elongation or other causes of upper airway obstruction.

**FIGURE 2:**
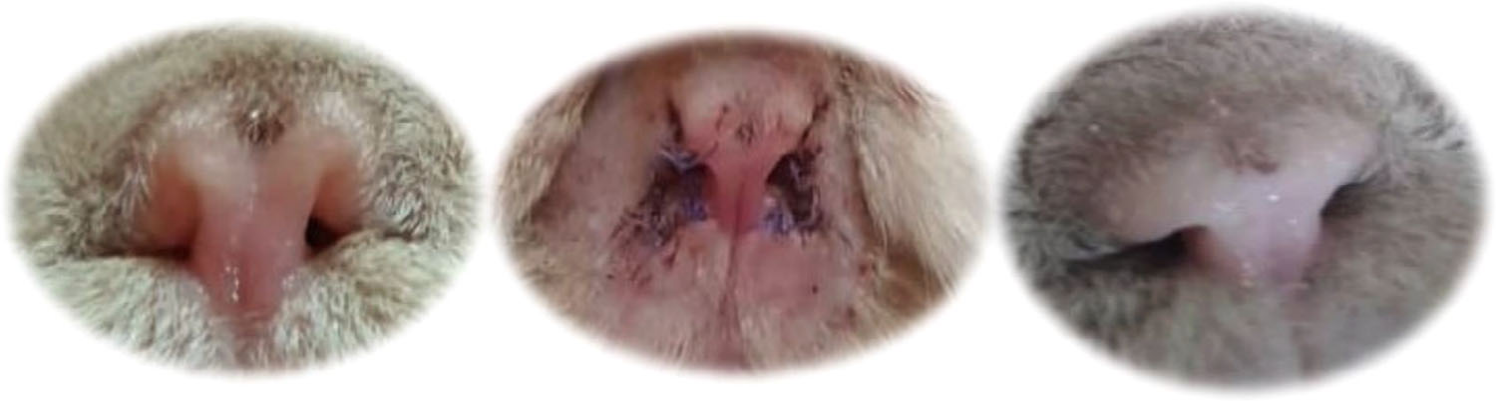
From right to left: One of the patients before surgery, 1 day after and 9 months after surgery.

In one of the cats, concurrent alar fold axial deviation was noted during the surgical procedure. Thus, in addition to the redundant skin resection, a rhinoplasty, including wedge resection, was also performed. After surgical treatment, the cases were reassessed the following day, and again at 2 weeks (Figure 2), 3 months and 9 months post‐surgery. No surgical complications were reported. The cats’ owners were very satisfied with the outcome and reported that stertorous breathing was reduced, or eliminated. No further respiratory distress was reported after surgery.

## 4 DISCUSSION

A brachycephalic phenotype is a potential cause of inspiratory problems in domestic animals, and, in cats, stenotic nares is the most commonly reported feature (Chanel N Berns et al., 2020; Sieslack et al., 2021).

Previous studies have suggested that this is predominantly a result of redundant skin in the ventral floor of the nose (Chanel N Berns et al., 2020). Farnworth et al., 2016. studied 239 cats in 2016. The owners were questioned regarding their pets’ respiratory noise, level of activity and physical characteristics and they were asked to submit images of their cats. The results showed that exercise intolerance was clearly associated with the facial conformation of cats, such that a shorter muzzle and a smaller ‘nose position ratio’ (calculated by dividing the eye‐nose distance by the foreface length and multiplying by 100) were associated with respiratory difficulties and abnormal breathing sounds (Farnworth et al., 2016). In 2021, Jana et al. studied facial conformation characteristics in Persian cats and compared them to exotic shorthair cats. They identified that nasal airway cross‐sectional areas were significantly smaller in Persian cats (Sieslack et al., 2021).

It is important to recognize that, as in dogs, cats with stenotic nares may additionally suffer from other components of BOAS such as soft palate elongation or abnormal nasopharyngeal turbinates (Chanel N. Berns et al., 2020). In light of this, decisions regarding surgical intervention should only be reached after careful assessment of the patient to reach an accurate diagnosis. In this study, thorough physical examinations, diagnostic imaging and upper airway assessment under general anaesthesia were performed to exclude the presence of other significant BOAS‐related abnormalities prior to surgical intervention.

In 2021, Berns et al. concluded that, in cats exhibiting BOAS because of nares stenosis, a single pedicle advancement flap can be used as surgical treatment. The authors reasoned that an increase in airway cross‐sectional area would reduce resistance to airflow and optimize respiratory tidal volume. Poiseuille's law shows that halving the radius of a tube will increase the resistance to flow by 16 times, such that even a small narrowing of an airway may markedly increase respiratory effort (Sieslack et al., 2021). In corcordance with the findings of Bern's et al., the single pedicle advancement flap surgical technique was found to be successful in all three patients with very satisfactory long‐term outcomes.

The choice of surgical technique to treat stenotic nares is also dependent on the presence or absence of axial deviation of the alar wing, which may be a contributory factor. Indeed, this was found in one of three cats in our study and in one of five cats in the study by Berns et al., and in such cases, wedge alarplasty must be performed in addition to skin fold resection.

In conclusion, this case series further confirms that, by targeting the major anatomical cause of inspiratory obstruction in cats with stenotic nares, this simple surgical technique offers effective and sustained resolution of respiratory distress.

## AUTHOR CONTRIBUTIONS

Negin Rahimdoost Mozhdehi: methodology, validation, writing—original draft. H. Salari Sedigh: data curation, investigation, visualization, writing—review & editing. Hossein Kazemi Mehrjerdi: conceptualization, data curation, investigation, methodology, supervision, validation, visualization, writing—review & editing. All authors checked and approved the final version of the manuscript for publication in the present journal.

## CONFLICT OF INTEREST STATEMENT

The authors have no conflict of interest to report.

## ETHICAL STATEMENT

All applicable international, national and/or institutional guidelines for the surgery and postoperative care of the animals were followed. Full information was given to the owners and consent was obtained.

### PEER REVIEW

The peer review history for this article is available at https://www.webofscience.com/api/gateway/wos/peer‐review/10.1002/vms3.1309

## Data Availability

No.
